# Successful Treatment of Pemphigus Vulgaris With the Extensive Mucocutaneous Lesions in an Elderly Patient

**DOI:** 10.5812/ircmj.13967

**Published:** 2014-06-05

**Authors:** Mohsen Masjedi, Ali Asilian, Zabihollah Shahmoradi, Parvin Rajabi Dehnavi, Bahareh Abtahi Naeini

**Affiliations:** 1 Department of Immunology, Faculty of Medicine, Isfahan University of Medical Sciences, Isfahan, IR Iran; 2 Department of Dermatology, Faculty of Medicine, Isfahan University of Medical Sciences, Isfahan, IR Iran; 3 Medicine Students’ Research Committee, School of Medicine, Isfahan University of Medical Sciences, Isfahan, IR Iran

**Keywords:** Pemphigus, Aged, Therapeutics

## Abstract

**Introduction:**

Pemphigus vulgaris (PV) is a chronic and infrequent autoimmune mucocutaneous disease that is characterized by the loose blisters and erosions on the skin and mucous membrane. Middle-aged adults are affect most frequently and the elderly and juvenile cases are infrequent. Herein, we reported a case of pemphigus vulgaris in an elderly patient.

**Case Presentation:**

We reported a case of pemphigus vulgaris in a 79-year-old patient with the extensive mucocutaneous lesions. We also reviewed the literature in MEDLINE with keywords such as pemphigus vulgaris, elderly, mucocutaneous lesions, oral lesions, and treatment.

**Discussion:**

We have to stress that the importance of this case report is its presentation in an elderly patient, as the frequent age of presentation in the Iranian patients is the middle age. On the other hand, PV occurs rarely, thus, the reporting of any rare case with some exceptions is important.

## 1. Introduction

Pemphigus vulgaris (PV) is an autoimmune vesiculobullous skin disease in which the immune system produces autoantibody against the specific proteins of the desmosomal adhesion complex that lead to the intraepidermal blister formation (1). The pathogenesis of this disease is not fully understood yet (2). PV is an infrequent disease; its incidence changes from 0.5 to 3.2 cases per 100000 individuals. Basically, PV covers an extensive age range with the peak frequency between the third and sixth decades of life (3). Although PV most frequently affects middle-aged adults, we report the case of a 79-year-old elderly patient with the extensive PV lesions who remitted completely with a combination therapy of oral prednisolone and azathioprine. Thus, we have to stress the importance of this case report in the elderly, as the mean age of presentation in the Iranian patients is the middle age, and not the elderly (4). On the other hand, PV occurs rarely (5); thus, reporting any rare case with some exceptions is of great importance.

## 2. Case Presentation

**Figure 1.**
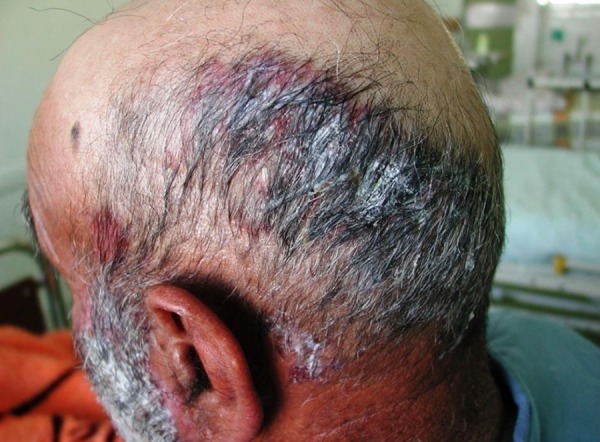
Pemphigus Vulgaris Lesions (Ulcer and Heavy Crust) on the Scalp

**Figure 2.**
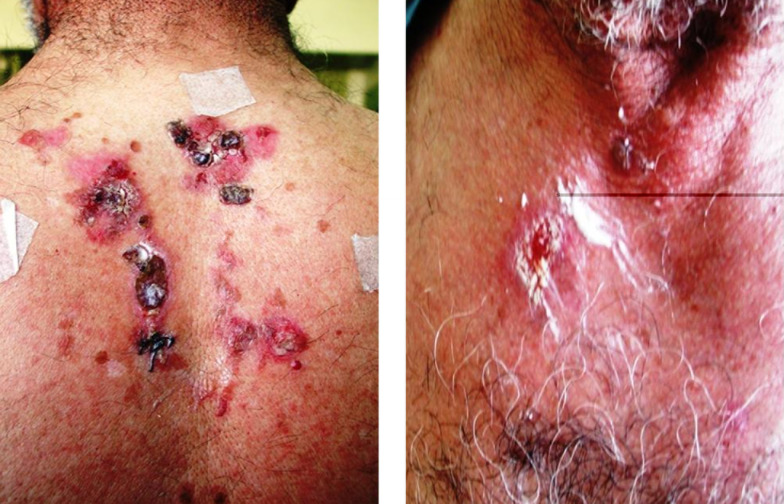
Pemphigus Vulgaris Ulcer and Hemorrhagic Crust on the Trunk

**Figure 3.**
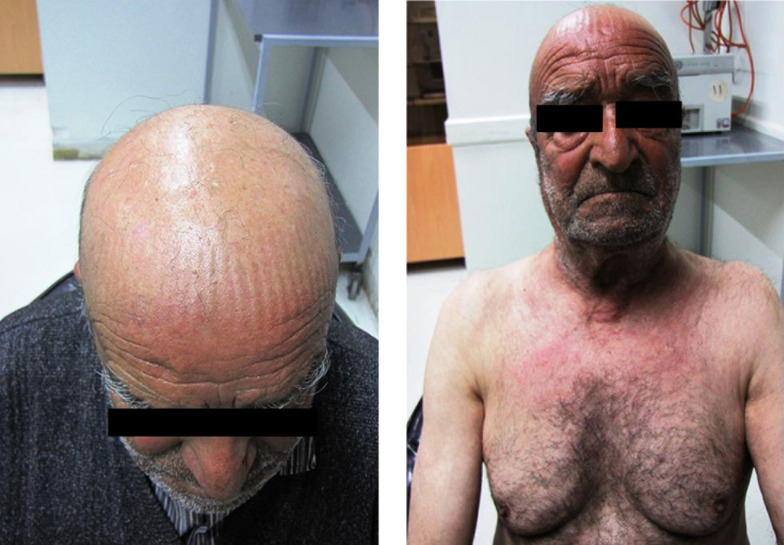
Pemphigus Vulgaris Following Successful Treatment Wound healing and tissue repair of the scalp and trunk in the patient following four-year successful treatment with prednisolone and azathioprine.

**Figure 4.**
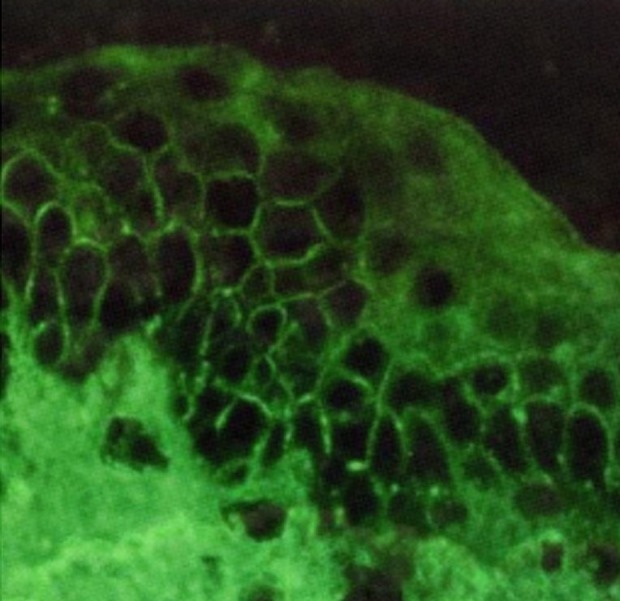
Direct immunofluorescence microscopy (DIF) of the cutaneous lesions revealed weak in vivo IgG deposition on the keratinocyte cell surface from the mid to upper epidermal layers (weakly positive lace like pattern in the epidermis, original magnification x100).

A 79-year-old man from Fereydunshahr Town, Isfahan Province, Iran, was referred to the Dermatology Clinic of Al-Zahra Hospital of Isfahan with the bullous lesions on his head. Two months before the development of these lesions, the patient had lesions on his trunk, face, and gingivae. Neither of the common sites in the oral cavity were affected except the gingivae. There was no evidence of tense blisters and itch. There was no history of urticarial lesions. He had no history of using any medication (Table 1). Intraoral examination showed erosions and desquamation with the erythematous areas on the gingivae, which were not healing. Crusted plaques were also seen on his scalp, face, and trunk (Figures 1 and 2). With the suspicion of PV, the biopsy specimen was taken from pemphigus lesions. Histopathological study of the skin lesion showed a suprabasal bulla containing some acantholytic cells. The basal cells rendered a tombstone appearance. In the dermis, there was a mild, superficial perivascular, lymphocytic infiltrate with a few eosinophils. The direct immunofluorescence (DIF) study also showed the characteristics deposition of IgG in the lace-like pattern. Hence, based on the clinical, histopathological, and immunofluorescence examination findings, the patient was diagnosed with PV (Table 1). Subsequently, he received systemic therapy with the oral prednisolone at an initial dosage of 70 mg/day (1 mg/kg/day), and azathioprine to reduce the inflammatory response and autoantibody production. He was advised to follow his treatment. Accordingly, the consent form was obtained from the patient and the study protocol conformed to the ethical guidelines of the 1975 Declaration of Helsinki as reflected in a prior approval by the Institution's Human Research Committee; the patient was followed for four years from October 2008 to October 2012. In October 2012, he was admitted to a local Clinic and the dose of prednisolone was slowly tapered down to 12.5 mg/day (a decrease of 5 mg every two weeks). Following physical examination, interestingly, the past lesions were completely healed (Figure 3) and it clearly demonstrated complete remission achieved four years from diagnosis. Accordingly, the skin biopsy was taken from the erythematous back, and the test showed the weak in vivo IgG deposition at the intercellular level in the stratum basale, stratum spinosum, and the basement membrane in the healed area of the skin (Figure 4). The patient remained under the treatment of 10 mg/day and 100 mg/day of prednisolone and azathioprine, respectively. He also received 1000-mg/day calcium and 400-IU/day vitamin D_3_ for the treatment of osteoporosis for the following two months.

**Table 1.** Different Variables of Pemphigus Vulgaris Reported in This Case ^a^

Variables	Description
**Age, y**	79
**Sex**	Male
**Patient's History**	No drug history, no urticarial lesions
**Clinical Findings**	Lesions on the scalp; ulcer and hemorrhagic crust on the trunk
**Mucosal Lesions**	Oral lesions: erosions and desquamation with the erythematous areas on the gingiva (desquamative gingivitis)
**Pathological Findings**	A suprabasal bulla containing some acantholytic cells. The basal cells rendered a “tombstone” appearance. In the dermis, a mild, superficial perivascular, lymphocytic infiltrate with a few eosinophils were seen.
**DIF and IIF**	IgG, in the dermis; deposition of IgG in the lace-like pattern
**Diagnosis**	Pemphigus vulgaris
**Treatment**	The initial low-dose 70-mg/d oral prednisolone, then tapered to 50 mg/d, subsequently a 5 mg reduction of prednisolone every two weeks led to complete remission of lesions, The patient remained under the treatment of 10mg/d and 100 mg/d of prednisolone and azathioprine, respectively.
**Complication**	Osteoporosis

^a^ Abbreviations: DIF, direct immunofluorescence; and IIF, indirect immunofluorescence.

## 3. Discussion

We reported a case of PV in an elderly patient with the extensive mucocutaneous lesions and his successful treatment. We have recently shown that the mean age of patients at PV onset is 40.19 years in the central provinces of Iran; the youngest patient in our study was 17 years old. Although Langan et al. have reported that the median age at presentation in the UK is 71 years old (range, 21-102) (6), our finding were consistent with the finding of Asilian et al. who have found the mean age at onset of 41.1 years in the central areas of Iran (4). On the other hand, Martel et al. reported that PV incidence displays two peak in the third and sixth decades of life (5). In addition, several other studies have shown that PV occurs most frequently at the fifth decade of life and not in the elderly (7, 8). Moreover, we must stress that the elderly people are prone to development of the blistering disorders, especially the autoimmune bullous disorders such as the bullous pemphigoid. Although the main reason is not fully known, it could be partially explained by the immunologic dysregulation that occurs with aging. Another potential reason is the differences in the skin of elderly patients in comparison with the younger adults’ skin. In fact, one study suggested that chemical induction of early blisters occurs more readily in the elderly skin in comparison with the younger adults’ skin (9).

PV is a serious disease that needs treatment as it is almost fetal if left untreated. Treatment consists of corticosteroids, gold-containing drugs, or immunosuppressor medications such as azathioprine and methotrexate. Plasmapheresis and the monoclonal anti-CD_20_ antibody may be used in addition to the systemic drugs to decrease the concentration of antibodies in the bloodstream (10). Unfortunately, the use of high-dose systemic corticosteroids is associated with complications such as osteoporosis and corticosteroid-induced hyperglycemia (11).

Prognosis is not very promising in the patients with the extensive PV and in the elderly patients. Our patient was an elderly man and used a combination of prednisolone and azathioprine for four years; however, he survived and only showed osteoporosis, which might be due to long-term consumption of prednisolone; however, aging is another important risk factor for osteoporosis. The osteoporosis in this patient would be due to a combination of prednisolone therapy and aging. In fact, there were two reasons for the complete remission of this patient. The first one was taking medication. The patient received the initial low-dose oral prednisolone at the concentration of 70 mg/day for ten days while he was hospitalized. Subsequently, the prednisolone dose was reduced to 50 mg/day until two months prior to readmission to the hospital. The reason for this decrease was minimizing the risk of potential side effects (3). Based on the clinical examinations, and laboratory tests, the patient experienced osteoporosis from his first visit (2008) through the follow-up visit (2012). He recently developed runny eyes; however, longer follow-up may reveal other side effects. As mentioned above, two months before readmission, a 5 mg reduction of prednisolone every two weeks was suggested by the local physician; however, a report has suggested a 50% reduction every two weeks once healing is induced and continued with healing of most lesions. In addition, Herman et al. have suggested a reduction of 5 to 10 mg of prednisolone per week and more slowly less than 20 mg prednisolone per day; however, the dosing schedules are vastly experimental and are adjusted on the practical experience and patients’ situation (3). This means that the patient should be followed up. Ultimately, the treatment may be withdrawn if there is prolonged clinical healing. 

The second probable reason was that the patient was very old and since the immune system is suppressed with age and PV is immune-mediated, we may also conclude that the disease has subsided in our case due to the age-related immune suppression.
